# Long-term, self-reported health outcomes in kidney donors

**DOI:** 10.1186/s12882-016-0221-y

**Published:** 2016-01-12

**Authors:** Käthe Meyer, Astrid Klopstad Wahl, Ida Torunn Bjørk, Torbjørn Wisløff, Anders Hartmann, Marit Helen Andersen

**Affiliations:** Department of Transplantation Medicine, Division of Cancer, Surgery and Transplantation, Oslo University Hospital, Rikshospitalet, Postbox 4950 Nydalen, 0424 Oslo, Norway; Department of Transplantation, Institute of Clinical Medicine, University of Oslo, Oslo, Norway; Department of Health Sciences, Institute of Health and Society, University of Oslo, Oslo, Norway; Department of Nursing Science, Institute of Health and Society, University of Oslo, Oslo, Norway; Oslo Centre for Biostatistics and Epidemiology, Research Support Services, Oslo University Hospital, Oslo, Norway; Department of Health Management and Health Economics, Institute of Health and Society, University of Oslo, Oslo, Norway

**Keywords:** Kidney donation, Quality of life, Fatigue, Questionnaires, Long term, Health outcomes

## Abstract

**Background:**

The wide use of healthy persons as kidney donors calls for awareness of risks associated with donation. Live kidney donation may impair quality of life (QOL) and result in fatigue. Long-term data on these issues are generally lacking in the donor population. Thus we aimed to investigate long-term self-reported health outcomes in a nationwide donor cohort.

**Methods:**

We assessed self-reported QOL, fatigue and psychosocial issues after donation in 217 donors representing 63 % of those who donated 8–12 years ago. QOL was measured using the generic Short Form-36 Health Survey (SF-36), fatigue using the Multidimensional Fatigue Inventory (MFI) and psychosocial issues using donor specific questions. For each of the 8 domains of SF-36 and the 5 domains of MFI, we performed generalized linear regression.

**Results:**

Donors scored high on QOL with mean scores between 63.9 and 91.4 (scale 1–100) for the 8 subscales. Recognition from family and friends was associated with higher QOL scores in four domains. There were no significant gender differences. Fatigue scores were generally low. Females generally scored higher than males on all five dimensions of fatigue, although significantly only on two. Recipient still alive was associated with lower scores on mental fatigue. Regretting donors scored higher than average on all domains of fatigue. Recipient death, worries about own health and worsened relationship with the recipient influenced willingness to donate in retrospect. Donor age did not affect long-term health outcomes.

**Conclusions:**

Eight till 12 years after donation QOL scores were generally high and improved with recogniton from family and friends. Fatigue was independent of donor age and more pronounced in females and in those who regretted donation.

## Background

End stage renal disease is an escalating health problem worldwide, with kidney transplantation being the gold standard treatment. Due to the increasing shortage of organs from deceased donors, transplantation with live kidney donor (LKD) has increased by 50 % [1], and in some countries, more than half of kidney transplants are live donor kidneys. The introduction of laparoscopic donor nephrectomy with a less traumatic surgery has boosted the use of LKD and also allowed for more extensive use of elderly donors. The wide use of healthy persons as kidney donors for the benefit of others calls for awareness to the risks associated with donation. Beyond the surgical and medical risks, there is evidence suggesting that live donation is associated with a decreased donor quality of life (QOL) and increased fatigue [2–4].

It seems to be a gender difference in who become live donors, and in several studies female donors outnumber male donors [3, 5–7]. Tumin and colleagues performed a comparison of QOL between donors and a control group [6]. They found a gender and age difference in scores among the donors and that male donors and donors older than 56 years had higher scores. A study comparing younger and elderly kidney donors showed that LKDs who were older than 60 years at the time of donation recovered faster in terms of QOL than younger donors who did not recover completely in all the domains within one year [8]. Importantly, long-term data on the associations between high donor age or gender differences are lacking.

Various factors other than age have also shown associations with QOL and fatigue post-donation, such as relationship to the recipient, pre-donation expectations, donation-related experience, transplant outcome and support from health professionals, family and friends [2, 3, 9, 10]. Furthermore, positive experiences and perceptions of support seem to be protective, while reduced QOL has been related to fatigue, pain, long recovery time and recipient graft-failure.

Altogether, the knowledge about long-term consequences of donor nephrectomy is sparse because most existing follow-up studies have a shorter time-span than 10 years [9, 11, 12], a variable time-span [3, 5, 13], and some have small samples [2, 6, 11, 14]. New data about long-term consequences after donation may provide a better basis for safe expansion of donor selection criteria [4, 15], informed consent [16, 17] and guidelines for long-term follow-up [18].

The present study is unique in the sense that this investigation uses a nationwide cohort that has been followed for 8–12 years. The aims were to investigate factors associated with long-term self-reported QOL and fatigue with particular reference to age groups.

## Methods

The study was approved by the Regional Medical Research Committee for Health South East of Norway (2011/2595 D) and the hospital’s data protection officer. The results are presented in such a way that no individual can be recognized in any publication or presentation of the data. This study was designed to investigate QOL and fatigue in LKDs representing all parts of Norway.

### Study design and population

**Fig. 1:**
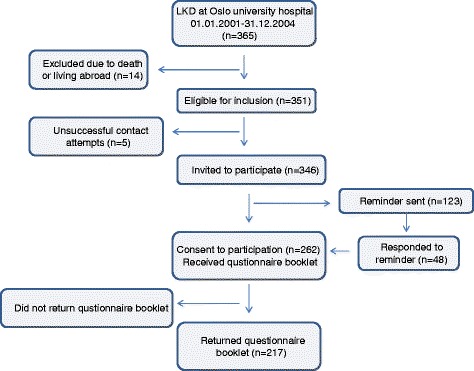
Inclusion criteria flow diagram. The diagram describes how the sample of living kidney donors (LKD) who completed the questionnaires for this study (*N* = 217) was derived from the total donors at Oslo university hospital in 2001–2004

A cross-sectional survey was performed using eligible Norwegian kidney donors (N = 351) who donated a kidney at Oslo University Hospital, the Norwegian transplant center, between 2001–2004. The eligible donors were invited by mail, including one reminder, to participate in the study. The invitation letter included information about the purpose of the study and ensured confidentiality. To be as close as possible to 10 years follow-up for all LKD, those who donated in 2001 and 2002 received the invitation in November 2012 and LKD who donated in 2003 and 2004 were invited in May 2013. Figure 1 shows a diagram of the inclusion criteria; 262 donors who agreed to participate were sent the questionnaire booklet, and informed consent to partake in the study was given by the 217 donors who returned the questionnaire.

### Questionnaires

#### QOL

To measure QOL, we used the generic Short Form-36 Health Survey (SF-36v2®) [19]. The instrument includes 36 items and evaluates a physical (PCS) and mental (MCS) component score in addition to eight different domains: physical functioning (PF), role physical (RP), vitality (VT), mental health (MH), role emotional (RE), social functioning (SF), general health (GH), and bodily pain (BP). Each of the eight subscales has a theoretical range of 0–100, and lower scores indicate reduced QOL. The instrument has been translated into and validated in Norwegian [20] and has been used in several studies among LKD [2, 3, 14, 21].

### Fatigue

The Multidimensional Fatigue Inventory (MFI) (22) was used to measure fatigue, which includes 20 items covering the following five dimensions: general fatigue (GF), physical fatigue (PF), mental fatigue (MF), reduced motivation (RM) and reduced activity (RA). Each subscale has a theoretical range of 4–20, and a higher score indicates more fatigue. MFI has been translated into Norwegian and validated [22] and is used in other studies to measure fatigue after kidney donation [2, 11, 14, 23].

### Donor specific questions

The participants also responded to donor specific questions, measuring psychosocial and clinical factors, such as regret of donation (yes/no/do not know), recipient outcome (from much better than expected to much worse than expected), economic problems (from to a great extent to not at all), regular follow-up (from annually to never), recognition from family, friends and health professionals (from to a great extent to not at all), use of analgesics or tranquilizers/ hypnotics (from daily to never) and satisfaction with life before and after donation (from very satisfied to very dissatisfied) [21].

### Demographic variables

Demographic variables included age, gender, marital status, educational level, vocational status and relationship to the recipient.

### Statistical analyses

The descriptive data are presented with frequencies and percentages. Due to the skewed distribution of QOL and fatigue, we used the non-parametric independent samples Wilcoxon Mann-Whitney *U*-test to test for differences in QOL and fatigue between males and females, and elderly and younger donors (<60 years or ≥ 60 years at donation time). To examine factors that may be associated with QOL and fatigue 10 years post-donation we used generalized linear model with gamma family and log link, as default. For regressions where gamma family and log link was not appropriate, we used Poisson with log link or gamma with identity link. Independent variables were recipient outcome, feeling responsibility for recipient’s health, recognition from family and friends, and health professionals’, use of analgesics and tranquilizers/hypnotics, and donor’s relationship to the recipient. Covariates were recognized demographics, such as age groups, gender, marital status and vocational status. The generalized linear model with Poisson family was used to investigate any association between the abovementioned factors and overall satisfaction with life 10 years after donation. One way ANOVA was used to examine the relationship between feeling responsible for the recipient’s health and expectations for the recipient’s health. Missing data were treated according to the manual for SF-36v2 ® [24] and for fatigue and donor specific questions treated as missing. A two-tailed *p*-value less than 0.05 was regarded as statistically significant. All analyses were performed using the statistical package for social sciences version 21 (SPSS Inc. Chicago, IL, USA). Testing for appropriate link function and family for generalized linear regression was done with linktest in STATA 13.1 (StataCorp, College Station, Texas, USA).

## Results

### Demographics and relationship to the recipient

The response rate was 63 % and median follow-up time was 10 years (range 8.5–12 years). Table 1 shows the characteristics of the donors by age group, older and younger than 70 years at the time of follow-up (60 years at donation). Overall, the mean age of the donors at follow-up was 59.5 years (range 31–91) and 34 (15.7 %) of the donors were 70 years or older. The majority (63.6 %) were females, and the most common relationship to the recipient was sibling (33.2 %) followed by parent (24.4 %). Parents were more represented in the elderly group, and more elderly donors were widowed and retired compared to younger donors. No new information was found about associations between self-reported QOL and fatigue, and the covariates marital status, educational level and vocational status

**Table 1:** Donor demographics and relationship to recipient by age groups

	All donors		<70 years		>70 years		
	*n*		*n*		*n*		*p*
Donors	217	(100 %)	183	(84.3 %)	34	(15.7 %)	
Gender							0.59
Female	138	(63.6 %)	115	(62.8 %)	23	(67.6 %)	
Male	79	(36.4 %)	68	(37.2 %)	11	(32.4 %)	
Marital status							0.04*
Single/divorced/widowed	52	(24 %)	39	(21.3 %)	13	(38.2 %)	
Married/cohabitant	164	(75.6 %)	143	(78.1 %)	21	(61.8 %)	
Educational level							0.88
High school or less	120	(55.3 %)	101	(55.2 %)	19	(55.9 %)	
College or university	93	(42.9 %)	79	(43.2 %)	14	(41.2 %)	
Vocational status							0.00*
Employed	147	(67.7 %)	139	(76 %)	7	(20.6 %)	
Not employed	70	(32.3 %)	42	(23 %)	27	(79.4 %)	
Donor’s relation to recipient							0.04*
Offspring	23	(10.6 %)	22	(12.0 %)	1	(2.9 %)	
Parents	53	(24.4 %)	39	(21.3 %)	14	(41.2 %)	
Sibling	73	(33.6 %)	64	(35.0 %)	8	(23.5 %)	
Spouse	37	(17.1 %)	29	(15.8 %)	8	(23.5 %)	
Other	31	(14.3 %)	27	(14.8 %)	3	(8.8 %)	

Pearson’s chi square: *P*-value is between age groups, asymp 2-tailed, *p < 0.05

Educational level comprise 213 donors, 4 unknown

### QOL

QOL scores evaluated by the SF-36 questionnaire are shown in Table 2. Females scored significantly lower in the domains RP and RE. There were no differences between donors <70 years or donors ≥70 years of age at follow up.

**Table 2:** QOL scores by age and gender

SF-36 Subscales		Total sample	Age groups			Gender		
Mean (SD)	*n*		<70y	≥70y	*P*	Male	Female	*P*
Physical function	208	89.7 (15.7)	90.1 (15.2)	86.1 (19.2)	0.29	92.4 (12.1)	87.8 (17.6)	0.13
Role physical	214	86.3 (24.2)	85.8 (24.8)	86.6 (25.1)	0.36	91.8 (20.7)	82.4 (26.4)	0.01*
Bodily pain	216	76.8 (26.0)	76.8 (26.2)	79.1 (25.7)	0.76	81.5 (22.9)	74.5 (27.5)	0.11
General health	213	80.5 (21.0)	81.6 (20.4)	78.5 (22.2)	0.16	85.6 (15.8)	78.6 (22.7)	0.12
Vitality	214	63.9 (22.9)	64.3 (23.4)	62.0 (23.3)	0.38	68.2 (22.2)	61.5 (23.7)	0.11
Social function	214	88.9 (20.2)	89.4 (19.7)	86.1 (23.1)	0.23	91.9 (16.5)	87.2 (21.9)	0.17
Role emotional	216	91.4 (17.0)	91.6 (17.2)	89.2 (19.3)	0.13	95.3 (10.9)	88.9 (20.1)	0.03*
Mental health	216	82.6 (16.1)	82.7 (16.2)	82.0 (15.9)	0.43	84.9 (15.1)	81.3 (16.7)	0.20

Non-parametric independent samples, Wilcoxon, Mann-Whitney U- test: Values are given as mean (SD), *P*-value is asymp. 2-tailed, *p < 0.05

### Fatigue

Results of measures of the different dimensions of fatigue tested with the MFI questionnaire are shown in Table 3. Females scored significantly higher than males on most scales. No differences in scores between donors < 70 years and ≥ 70 years of age were found.

**Table 3:** Fatigue scores by age group and gender

MFI Scale scores		Total sample	Age groups			Gender		
Mean (SD)	*n*		<70y	≥70y	*p*	Male	Female	*P*
General fatigue	207	8.8 (4.4)	8.8 (4.5)	8.4 (4.0)	0.89	7.7 (3.8)	9.3 (4.6)	0.01*
Physical fatigue	209	8.2 (4.2)	8.1 (4.1)	8.7 (4.5)	0.28	7.3 (3.6)	8.7 (4.4)	0.01*
Reduced activity	211	8.3 (4.1)	8.2 (3.9)	8.6 (4.3)	0.26	7.7 (3.5)	8.5 (4.2)	0.09
Reduced motivation	208	7.3 (3.1)	7.1 (3.0)	7.0 (2.6)	0.61	6.7 (2.9)	7.4 (3.0)	0.04*
Mental fatigue	212	8.0 (3.5)	8.0 (3.6)	8.1 (2.9)	0.25	7.4 (3.5)	8.3 (3.5)	0.03*

Non-parametric independent samples, Wilcoxon, Mann -Whitney *U*-test: Values are given as mean (SD), *P*-value is asymp. 2-tailed, *p < 0.05

### Donor specific questions

**Fig. 2:**
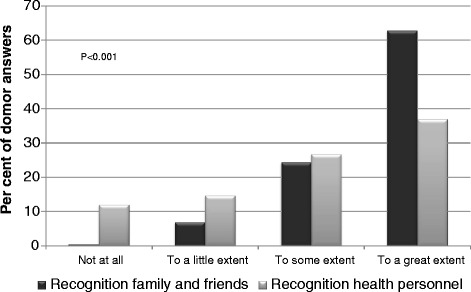
Responses to donor specific questions according to levels of agreement (per cent)

Nearly all LKDs (94 %) would have donated again; only seven (3.2 %) would not. Among those seven, there were donors who perceived that the donation had been harmful to own health, the relationship with the recipient had worsened, or reported the recipient had lost the graft or died. Thirteen LKDs (6 %) believed the donation had been harmful to his or her own health. Sixteen LKDs (7.4 %) reported economic problems related to the donation, nine (4 %) claimed loss of income was the reason, and the donation had caused a change in vocation for 11 donors (5 %). Twenty three LKDs (10.7 %) used tranquilizers/hypnotics and 31 (14.4 %) used analgesics daily or every week. Female donors were more frequent users than male donors; 13.9 % vs. 5.2 % and 19.7 % vs. 5.1 % respectively. More than half of the LKDs (54.4 %) did not see a nephrologist for medical follow-up in the long-term after donation, while 55 (25.3 %) had regular visits every year or every other year. As illustrated in Fig. 2, LKDs perceived more recognition from family and friends than from healthcare personnel (*p* < 0.001).

The majority (71.5 %) reported to perceive that the recipient’s health was better than or as they had expected. Responsibility for the recipients health was pereceived by nearly half of the donors, 35 % felt some responsibility and 12 % felt a great extent of responsibility for the recipient’s health. There was a relationship between the donor’s feeling of responsibility and the perceived health of the recipient at follow-up (*p* = 0.002). The feeling of responsibility was highest when perceived health of the recipient was much better than expected.

As shown in Table 4, the donors’ perception of recognition from family and friends was associated with the SF-36 dimensions RP, BP, VT and RE. There was no association between recipient outcome or the donor feeling responsible for recipient’s health and QOL.

**Table 4:** Associations between QOL scores (SF-36) and donor specific questions

		Physical function^a^	Role physical^b^	Bodily pain^b^	General health^a^	Vitality^b^	Social function^a^	Role emotional^a^	Mental health^a^
	*n*	159	164	166	163	163	167	166	166
Recognition									
Family and friends	B (st.error)	3.242 (2.004)	7.984* (4.029)	7.490* (3.153)	0.063 (0.041)	6.776* (3.077)	0.048 (0.039)	0.055* (0.028)	0.045 (0.030)
Health personnel	B (st.error)	0.342 (1.322)	1.527 (2.830)	2.890 (2.496)	0.001 (0.027)	0.949 (2.266)	0.010 (0.025)	0.009 (0.017)	0.014 (0.020)
Recipient’s outcome									
Still alive	B (st.error)	1.458 (4.705)	0.499 (9.913)	5.959 (8.205)	−0.027 (0.097)	0.363 (7.799)	0.036 (0.092)	0.034 (0.064)	0.075 (0.071)
Responsibility									
Feeling responsible for recipient’s health	B (st.error)	−0.612 (1.093)	3.277 (2.420)	1.271 (2.100)	0.008 (0.023)	0.754 (1.862)	0.020 (0.021)	0.021 (0.015)	0.003 (0.016)
Donor’s relation^c^									
Offspring	B (st.error)	−0.126 (4.556)	2.923 (9.282)	1.725 (8.156)	0.054 (0.094)	−4.816 (7.850)	−0.149 (0.089)	−0.066 (0.062)	−0.041 (0.070)
Parents	B (st.error)	0.021 (3.974)	8.459 (8.275)	8.052 (7.699)	0.068 (0.082)	−5.203 (7.059)	−0.104 (0.077)	−0.023 (0.054)	−0.001 (0.061)
Sibling	B (st.error)	−0.777 (3.553)	3.039 (7.351)	3.450 (6.724)	0.040 (0.074)	−0.410 (6.417)	−0.097 (0.069)	−0.001 (0.048)	0.016 (0.054)
Spouse	B (st.error)	−1.627 (4.201)	4.645 (8.266)	3.094 (7.602)	0.031 (0.084)	−5.367 (7.041)	−0.121 (0.080)	−0.052 (0.056)	−0.031 (0.062)
Use of analgesic^d^									
Daily/every week	B (st.error)	−8.034* (4.001)	−15.978 (8.639)	−20.299** (7.117)	−0.057 (0.081)	−4.438 (6.827)	−0.042 (0.077)	−0.096 (0.054)	0.005 (0.061)
Use of relaxant/ sleeping pills^e^									
Daily/every week	B (st.error)	−17.378** (4.817)	−11.263 (8.498)	−0.169 (8.631)	−0.116 (0.093)	−17.494** (6.437)	−0.095 (0.086)	−0.133* (0.060)	−0.099 (0.070)
Gender^f^									
Male	B (st.error)	4.355 (2.532)	6.980 (5.889)	3.118 (5.011)	0.079 (0.053)	4.853 (4.832)	0.044 (0.050)	0.045 (0.035)	0.035 (0.039)
Agegroup^g^									
<70 years	B (st.error)	4.419 (4.060)	3.637 (8.356)	7.450 (7.430)	0.070 (0.082)	5.565 (6.824)	0.079 (0.077)	0.076 (0.054)	0.020 (0.060)
Medical follow-up^h^									
No regular controls	B (st.error)	−2.743 (2.360)	−2.947 (4.985)	−0.842 (4.419)	−0.062 (0.049)	0.016 (4.417)	0.018 (0.046)	−0.028 (0.032)	−0.011 (0.036)

Generalized linear model: Each domain was analyzed separately; ^a^Gamma with log link, ^b^Gamma with identity; B, the regression coefficient; reference was: ^c^relation category friends/others, ^d^less than every week/never, ^e^less than every week/never, ^f^female, ^g^age ≥ 70 years, ^h^no controls; controlled for covariates marital status, educational level and vocational status; *p < 0.05, **p < 0.01

**Fig. 3:**
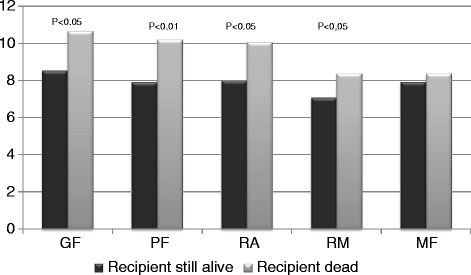
Donors’ mean scores on dimensions of fatigue when recipient was still alive or dead ten years after donation

Table 5 shows the associations between the dimensions of fatigue and donor specific questions and demographics. There were no associations between donor’s self-reported fatigue and responsibility for recipient health, recognition from others, or relationship to recipient at follow-up. As illustrated in Fig. 3 recipient’s death was significantly associated with higher scores in the MFI dimensions GF, PF, RA and RM.

**Table 5:** Associations between fatigue scores (MFI) and donor specific questions

		General fatigue^a^	Physical fatigue^b^	Reduced activity^a^	Reduced motivation^a^	Mental fatigue^a^
	*n*	163	163	163	161	164
Recognition						
Family and friends	B (st.error)	−0.021 (0.080)	0.066 (0.425)	−0.003 (0.058)	0.038 (0.049)	−0.016 (0.054)
Health personnel	B (st.error)	−0.038 (0.038)	−0.130 0.264	0.010 (0.039)	−0.012 (0.030)	−0.038 (0.034)
Recipient’s outcome						
Still alive	B (st.error)	0.113 (0.141)	−0.172 (1.060)	0.011 (0.133)	0.086 (0.110)	0.363** (0.126)
Responsibility						
Feeling responsible for recipient’s health	B (st.error)	0.051 (0.033)	0.095 (0.238)	0.002 (0.032)	−0.008 (0.026)	−0.015 (0.030)
Donor’s relation^c^						
Offspring	B (st.error)	−0.025 (0.135)	0.952 (0.895)	0.218 (0.130)	0.023 (0.107)	0.110 (0.122)
Parents	B (st.error)	−0.005 (0.118)	0.660 (0.763)	0.103 (0.114)	−0.001 (0.092)	−0.047 (0.106)
Sibling	B (st.error)	0.064 (0.104)	0.832 (0.653)	0.140 (0.099)	0.094 (0.082)	0.040 (0.094)
Spouse	B (st.error)	0.135 (0.126)	0.366 (0.809)	0.208 (0.119)	0.067 (0.099)	0.091 (0.111)
Use of analgesic^d^						
Daily/ every week	B (st.error)	0.356** (0.115)	3.599** (1.168)	0.258* (0.110)	0.071 (0.094)	0.158 (0.104)
Use of relaxant/ sleeping pills^e^						
Daily/ every week	B (st.error)	0.261* (0.128)	2.552* (1.277)	0.321* (0.125)	0.363*** (0.103)	0.161 (0.116)
Gender^f^						
Male	B (st.error)	−0.196* (0.078)	−1.139* (0.489)	−0.037 (0.072)	−0.072 (0.060)	−0.068 (0.068)
Agegroup^g^						
<70 years	B (st.error)	−0.088 (0.117)	−1.298 (0.889)	0.017 (0.114)	0.016 (0.092)	−0.093 (0.103)
Medical follow-up^h^						
No regular controls	B (st.error)	−0.009 (0.071)	0.299 (0.476)	−0.006 (0.068)	−0.009 (0.055)	−0.078 (0.063)

Generalized linear modell: each domain was analyzed separately; ^a^Gamma with log link, ^b^Gamma with identity; B, the regression coefficient; reference was: ^c^relation category friends/others, ^d^less than every week/never, ^e^less than every week/never, ^f^female, ^g^age ≥ 70 years, ^h^no controls; controlled for covariates marital status, educational level and vocational status; *p < 0.05, **p < 0.01, ***p < 0.001

Overall satisfaction with life 10 years after donation was positively associated with perceived recognition from health personnel (*p* < 0.01) and negatively associated with donors being younger than 70 years (*p* < 0.001).

LKDs who would not have donated again had higher mean scores than the average on all domains of MFI; GF (12.9), PF (13.4), RA (11.4) RM (11.0) and MF (10.4).

## Discussion

This study shows that the donors generally perceive their QOL as good, and also report a low degree of fatigue at 10 years after donation. The results are in line with studies reporting a good QOL in the short-term after transplantation [9, 11, 12]. However, in a follow-up study 10 years after donation Klop et al. reports both excellent health and a deviation from baseline values in several domains of QOL and fatigue [14]. The authors argue that the difference may be explained by an increase in age of 10 years. In the present survey representing a nationwide selection of donors followed according to European recommendations [18, 25] the research design did not allow for baseline data or a control group. However, approximately one third of the participants in this survey also participated in a Norwegian randomized study comparing laparoscopic and open donor nephrectomy [21]. The good health reported in the present study appears to be sustained long-term. Thus our study provides new knowledge about the long-term self-reported health outcomes that may have implications concerning information provided to future donors.

In the present study, a significant finding was the difference between males and females in the MFI for all domains except RA, and in the domains RP and RE in the SF-36. The gender difference in self-reported fatigue in our study differs from the results in a study on fatigue and physical function in mid-life [26]. Boter and colleagues found a strong association between physical function and the subscales in MFI but no gender difference. However, gender difference in fatigue is inconsistent in previous studies. While a study on the German population demonstrated a significant difference [27], the gender difference was present but not significant in a Swedish study [28], and a Danish study showed that while there was no general gender difference there seemed to be an excess of females with high scores [29]. Only few studies [2, 11, 14, 23] have specifically investigated fatigue after live donation, and as far as we know, none have examined the gender difference. Our results indicate that females may experience fatigue after donation. Since the majority of the donors are female it seems important to include information about fatigue in pre-donation information.

Another particular aspect of the present study was the effect of donation on the elderly population because elderly donors are frequently used. We defined an elderly donor as older than 60 years of age at the time of donation (70 years at follow-up) for comparison with previous studies on elderly donors [7, 30]. Even though there has been reported a linear relationship between dimensions of fatigue in MFI and age in a general population [27], we found no difference in self-reported fatigue between younger and older donors in the long-term. Nor did we find a difference in self-reported QOL. These results are in line with results from short-term observations [7, 30] and confirm the results from the long-term RELIVE study [3]. The elderly donors in our study also scored higher than the elderly participants (more than 70 years) in the Norwegian general population [20]. Although we have to have in mind that the normative data are nearly 20 years old, the long-term self-reported health seems not to be impaired in elderly donors. These results are reassuring for both clinicians and elderly persons considering being a kidney donor.

The large majority of the donors did not regret donation and would be willing to donate again as has been reported in many follow-up studies [11, 12, 31]. However, a small minority would say no if they were asked again, and the higher scores in fatigue in these donors call for attention. Fatigue among donors who regret donation has not been studied before and needs further investigation. We found that some of those who regretted donation had experienced recipient death. Our data also showed an association between fatigue and recipient death. This is in line with previous research which has demonstrated that recipient death can produce a feeling of guilt [32] and influence LKD’s well-being [10, 33]. In addition, there were donors who had experienced a negative change in the relationship with the recipient, which may be disappointing. Furthermore, three of those who regretted donation perceived that the donation had been harmful to his or her health. Worrying about own health may have provoked or contributed to the perception of fatigue. The real challenge would be to identify donors at risk for regret during donor work-up. Unfortunately, this aspect could not be addressed in the present study but paying attention to those who donate to a recipient at high-risk may be appropriate. Adverse outcomes for the recipient have also been found to be associated with adverse psychosocial outcomes for LKD in previous studies [10, 32, 33]. However, for the donor population as a whole, the donors in our study did not blame themselves if the result was poor. Nevertheless, recipient outcome better than expected and recipient still alive was associated with a positive emotional outcome and seemed to generate more responsibility among the donors.

Both live and deceased donation is generally acknowledged to be a good deed. Appreciation and recognition of the deed they have accomplished from the social environment in addition to improved recipient health may provide improved self-esteem and personal growth, which again may be associated with QOL and satisfaction with life. Findings from previous studies [32, 34, 35] such as changed roles, improved self-esteem, personal growth, and improved QOL among donors support this conclusion.

While Tong et al. [32] found that donors worry about potential kidney failure, physical well-being and ill health, a minority in our study considered the donation to be harmful to his or her health. The low number of donors who had regular visits to a nephrologist may both reflect that LKDs are healthy persons going through an extensive work-up ahead of the donation and that most recover within the first year post-donation. However, to obtain knowledge about the long-term consequences and ensure quality and safety, regular follow-up is essential [18, 36]. In Norway, which is a fairly small country and has limited mobility within the population, the possibility for medical follow-up for most donors may be better than in vast countries such as the USA [3, 36]. Still, less than half of the donors in the present study had medical follow-up 10 years after donation.

The strength of the present study is the sample size with a fairly high response rate 8–12 years after donation and representation from all parts of Norway [37]. The demographics and characteristics of the non-responders are similar to that of the responders. The data sets also had few missing data. We have used well-established methods previously validated in Norwegian populations. A limitation of cross-sectional follow-up studies is the lack of baseline data and a control group. Consequently, we do not know if there has been a change in self-reported outcomes 8–12 years after donation. Additionally, we do not have any information about the motivations to donate. There might be a recall bias in self-report up to 12 years later. Another limitation in this study is that we did not link our data to a recipient registry. However, we tried to restrict the weakness by questions about donors’ expectations and recipient outcome. We have contributed to new knowledge as seen from the donors’ perspective which recently was highlighted in the report from Thiessen et al (2015) as essential in the care of LKD.

## Conclusions

The long-term QOL of donors was reported as good both in younger and older donors and improved with recognition from family and friends. Female donors had higher scores on fatigue than male donors. A few donors regretted donation and those donors reported a high level of fatigue. Identifying and following donors who are at risk for regretting donation is important. More research is needed on gender differences and factors that are associated with fatigue after live kidney donation in the long-term compared to baseline data.

## Abbreviations

ANOVA: Analysis of variance
BP: Bodily pain
GF: General fatigue
GH: General health
LKD: Live kidney donors
MCS: Mental component score
MF: Mental fatigue
MFI: Multidimensional Fatigue Inventory
MH: Mental health
PCS: Physical component score
PF: Physical functioning
PF: Physical fatigue
QOL: Quality of life
RA: Reduced activity
RE: Role emotional
RM: Reduced motivation
RP: Role physical
SF: Social functioning
SF-36: Short Form-36 Health Survey
VT: Vitality
